# Racial Discrimination and Uptake of Dental Services among American Adults

**DOI:** 10.3390/ijerph16091558

**Published:** 2019-05-04

**Authors:** Wael Sabbah, Aswathikutty Gireesh, Malini Chari, Elsa K. Delgado-Angulo, Eduardo Bernabé

**Affiliations:** 1 Faculty of Dentistry, Oral & Craniofacial Sciences, King’s College London, Bessemer Road, London SE5 9RS, UK; wael.sabbah@kcl.ac.uk (W.S.); elsa.delgado_angulo@kcl.ac.uk (E.K.D.-A.); 2 Institute of Child Health, Department of Population, Policy and Practice, University College London, 30 Guilford St, London WC1N 1EH, UK; a.aswathikutty-gireesh@ucl.ac.uk; 3 Faculty of Dentistry, University of Toronto, 124 Edward St, Toronto, ON M5G 1G6, Canada; malini.chari@mail.utoronto.ca

**Keywords:** dental health services, social determinants of health, racism, socioeconomic factors

## Abstract

This study examined the relationship between racial discrimination and use of dental services among American adults. We used data from the 2014 Behavioral Risk Factor Surveillance System, a health-related telephone cross-sectional survey of a nationally representative sample of adults in the United States. Racial discrimination was indicated by two items, namely perception of discrimination while seeking healthcare within the past 12 months and emotional impact of discrimination within the past 30 days. Their association with dental visits in the past year was tested in logistic regression models adjusting for predisposing (age, gender, race/ethnicity, income, education, smoking status), enabling (health insurance), and need (missing teeth) factors. Approximately 3% of participants reported being discriminated when seeking healthcare in the past year, whereas 5% of participants reported the emotional impact of discrimination in the past month. Participants who experienced emotional impact of discrimination were less likely to have visited the dentist during the past year (Odds Ratios (OR): 0.57; 95% CI 0.44–0.73) than those who reported no emotional impact in a crude model. The association was attenuated but remained significant after adjustments for confounders (OR: 0.76, 95% CI 0.58–0.99). There was no association between healthcare discrimination and last year dental visit in the fully adjusted model. Emotional impact of racial discrimination was an important predictor of use of dental services. The provision of dental health services should be carefully assessed after taking account of racial discrimination and its emotional impacts within the larger context of social inequalities.

## 1. Introduction

Racial inequalities in use of dental services and their potential implications are widely documented in the literature [1,2,3,4,5,6]. These inequalities are mostly attributed to socioeconomic and cultural/behavioral attributes [7,8,9], but the question as to how the observed racial gaps in utilization of dental services could be a consequence of racial discrimination is not fully explicated [10,11].

Racial discrimination in healthcare is an individual’s appraisal of unfair treatment in a medical setting based on race, colour or national origin [12,13]. Discrimination can occur at individual, institutional, and structural levels [14]. It can operate through different mechanisms, subsequently leading to poor health outcomes and underutilization of services [13,15]. First, the institutional or structural racism arising from racially discriminatory unfair policies and institutional culture can lead to differential educational/employment opportunities, and thus, access to health-promoting resources [15]. Second, racism often leads to the development of implicit racial bias and explicit racial stereotypes, influence clinicians’ behavior decision making and communication process, and thus contribute to the differential treatment of members of same institution [16,17]. Third, racism can act as a psychosocial stressor that operates through physiological, psychological, and behavioral pathways which could have consequences for health [16]. Moreover, negative experiences have the potential to influence quality of healthcare, interpersonal trust, medical adherence levels, and treatment delays [18,19]. Finally, the racial discrimination deeply entrenched within the healthcare settings may alter the patient’s perception of healthcare interaction and the pattern of their healthcare access [20]. To be more precise, internalization of unfair treatment may engender involuntary responses such as anxiety or increased vigilance and voluntary coping responses like disengagement from situations or environments that negatively stereotype people [21]. This may possibly inhibit certain individuals from using a wide range of needed health services, including dental services.

Few studies have evaluated the impact of racial discrimination on oral health, with conflicting evidence. While racial discrimination was associated with experience of toothache among pregnant Aboriginal Australians [22], self-reported dental problems among Canadian immigrants [23] and tooth loss among pregnant Aboriginal Canadians [24], no association was found with periodontitis among Hispanic Americans [25], tooth loss among Brazilian civil servants [26], or oral health-related quality of life among pregnant Aboriginal Canadians [24]. Evidence on the impact of racial discrimination on utilization of dental services is even more limited. Racial discrimination was not associated with a dental visit within the past year among pregnant Aboriginal Canadians, but it was associated with being asked to pay for dental services by dental care providers despite entitlement to free dental care and seeking care off-reserve or out of the community [24]. Among pregnant Aboriginal Australians, racial discrimination was associated with having never visited a dentist before. However, no adjustment for participants’ socioeconomic status was attempted [27]. A broader implication of the negative impact of discrimination comes from qualitatively research, which showed that disadvantaged caregivers of Medicaid-enrolled children cited discriminatory behavior attributed to racism as one of the key barriers to accessing dental services [28,29].

Using the well-established Andersen’s behavioral model of health services use [30,31] as a theoretical framework to understand factors affecting an individual’s decision to use dental services, this study explored the relationship between racial discrimination and use of dental services among American adults. It was hypothesized that individuals experiencing racial discrimination would be less likely to use dental services than those without such experiences.

## 2. Materials and Methods

### 2.1. Data Source

The Centers for Disease Control and Prevention’s Behavioral Risk Factor Surveillance System (BRFSS) is an annual, state-based, random-digit-dialled, telephone health survey of the non-institutionalized US civilian population, aged 18 years or older, living in all 50 States, the District of Columbia, Puerto Rico, and Guam. In each geographic region, the BRFSS uses a disproportionate stratified sampling for the landline telephone survey and random sampling for the cellular telephone survey. For the landline telephone survey, interviewers collect data from a randomly selected adult in every participating household. For the cellular telephone survey, interviewers collect data from adults residing in a private residence or college housing who have a working cellular telephone. The data were collected using the BRFSS standardized questionnaire via computer-assisted telephone interviews (CATI). The BRFSS questionnaire consists of core, optional, and state-added questions modules. Interviewers were trained as per the BRFSS protocol, and confidentiality of participants was maintained by ensuring their anonymity to the interviewers [32].

We used data from the 2014 BRFSS because this was the most recent survey, including questions on both oral health and racial discrimination. In 2014, 464,664 interviews were made, representing a weighted median response rate of 47% (48.7% and 40.5% for landline and cellular telephones, respectively). Response rates varied from 25.1% to 60.1% across states.

The present analysis is limited to participants in the three states (Minnesota, Mississippi, and New Mexico) that included the optional module on reactions to race in 2014. There were 23,272 participants who completed the optional module on reactions to race and the core module on oral health. After exclusions due to missing values on covariates, the study sample include 11,950 adults.

### 2.2. Selection of Variables

The outcome variable of interest was utilization of dental services, which was assessed with the following item: “How long has it been since you last visited a dentist or a dental clinic for any reason?” with multiple response options (within the past year, 1 year but less than 2 years ago, 2 years but less than 5 years ago, 5 or more years ago and never). Responses were re-coded into those who visited a dentist in the past one year and those who did not visit one (reference group).

Two main explanatory variables indicating discrimination were taken from the reactions to race module of the questionnaire. First, perceived racial discrimination while seeking healthcare was assessed with the following question: “Within the past 12 months when seeking healthcare, do you feel your experiences were worse than, the same as, or better than people of other races?” with multiple response options (worse than other races, the same as other races, better than other races, worse than some races, better than others, only encountered people of the same race). For analysis, ‘worse than other/some races’ responses were coded as having experienced discrimination. The second indicator of discrimination was emotional impact of discrimination, which was assessed using the question: “Within the past 30 days, have you felt emotionally upset, for example angry, sad, or frustrated, as a result of how you were treated based on your race?” with yes/no response options.

Based on Andersen’s behavioral model of health services use [30,31], several covariates were included as potential confounders of the association between racial discrimination and use of dental services. They were predisposing (age, gender, race/ethnicity, income, education, and smoking), enabling (health insurance), and need factors (number of missing teeth). Self-identified race/ethnicity was categorized into five groups: Non-Hispanic White, non-Hispanic Black, Hispanic, and Asian/Other, and Multiracial. Yearly income was categorized into four groups: Less than $20,000, $20,000 to $34,999, $35,000 to $74,999, and $75,000 or more. A binary variable created for health insurance coverage was based on whether participants were enrolled in a healthcare plan (including Medicare and Medicaid). Educational attainment was categorized as less than high school, high school or equivalent, some college, and college graduate. Current smokers were respondents who reported smoking at least 100 cigarettes during their lifetimes and reported smoking “every day” or “some days”. Former smokers were those respondents who reported ever smoking at least 100 cigarettes but reported smoking “not at all”. Never smokers were respondents who reported smoking fewer than 100 cigarettes during their lifetimes. The original categories of missing teeth (none; 1 to 5; 6 or more, but not all; and all) were categorized into missing teeth and no missing teeth.

### 2.3. Statistical Analysis

Analyses were weighted to produce nationally representative estimates and accounted for the sampling design (clustering and stratification). Only cases with complete data in all variables were included in the analysis. Analyses were performed in Stata 14 (StataCorp, College Station, TX, USA).

We first described the characteristics of the sample according to predisposing (demographic factors, socioeconomic position, smoking status), enabling (health insurance), and need factors (missing teeth). Both indicators of racial discrimination were also included. Secondly, the proportion of individuals who had a dental visit in the past year were compared between categories of the two indicators of racial discrimination and covariates using the Chi-squared test.

Thereafter, we constructed two logistic regression models to assess the relationship between each indicator of racial discrimination (racial discrimination when seeking healthcare and emotional impact of racial discrimination) and utilization of dental services in the past year (outcome). Odds ratios (OR) were used as the measure of association. We first tested the association between racial discrimination when seeking healthcare and use of dental services in a crude model, which was subsequently adjusted for predisposing (sex, age, race/ethnicity, education, income, and smoking status in Model 1A), enabling (health insurance in Model 1B), and need (number of missing teeth in Model 1C) factors. No statistical interactions were included in the models. The same set of models was developed when testing the association between the emotional impact of racial discrimination and use of dental services (labelled as Models 2A to 2C).

## 3. Results

The characteristics of the study sample are presented in Table 1. The percentage of dental visits within the past year was 68.3% (95% CI 67.1–69.5). In addition, 2.7% (95% CI 2.3–3.1) of adults reported healthcare discrimination and 5.0% (95% CI 4.5–5.6) reported emotional impact of discrimination. Dental visits within the past year were more common among women, older, non-Hispanic White, and non-smoking participants. Those with more education, greater income, health insurance, and no missing teeth were also more likely to have visited the dentist in the last year.

Dental visits were less common among those who reported racial discrimination while using the healthcare system or reported emotional impact of discrimination than those who did not (Table 2). Those who experienced racial discrimination when seeking healthcare were less likely to have visited the dentist in the past year than those who did not have such an experience (OR: 0.57, 95% CI 0.41–0.79). Similarly, those who experienced the emotional impact of discrimination were less likely to have visited the dentist in the past year than their counterparts (OR: 0.57, 95% CI 0.44–0.73).

The association between healthcare discrimination and use of dental services was fully attenuated after adjustment for predisposing factors (demographics, socioeconomic position, and smoking status) and remained unchanged after subsequent adjustments for enabling (health insurance) and need (missing teeth) factors (Table 3). In the fully adjusted model, the odds ratio was 0.88 (95% CI 0.62–1.25). By contrast, the association between emotional impact of discrimination and use of dental services remained significant after adjustments for predisposing, enabling, and need factors. In the final model, those who experienced the emotional impact of racial discrimination were 25% (OR: 0.75, 95% CI 0.58–0.99) less likely to have visited the dentist in the past year than those without such experience.

## 4. Discussion

This study supported our hypothesis that racial discrimination is negatively associated with the utilization of dental services. Those who experienced the emotional impact of racial discrimination were less likely to have used dental services within the past year. This finding was robust to multiple adjustments for known determinants of utilization of dental services, which were carefully chosen according to the Andersen’s behavioral model of health services use [30,31].

Most of the current research on racial/ethnic inequalities in use of dental services gives scant attention to the role of racial discrimination by which this association might exist [11]. In this study, both healthcare discrimination and the emotional impact of discrimination were negatively associated with dental visits in crude regression models. Interestingly, the association between healthcare discrimination and dental visits was fully accounted for by controlling for predisposing factors, which emphasizes the role of socioeconomic position in explaining such an association. Having greater financial resources might give individuals more options of healthcare providers and, as such, prevent discriminatory experiences. 

The above finding also suggests two related points. One is that it might be easier to report emotional impacts of racial discrimination than actual experiences of discrimination. As with other subjective experiences, racial discrimination could be misperceived or overlooked, which can lead to underestimating or overestimating their actual occurrence. The other is that the emotional impact of discrimination can arise from a broader set of life experiences, not only those related to seeking healthcare. This argument explains why the prevalence of the emotional impact of discrimination was higher than that of healthcare discrimination despite the former having a shorter recall frame than the latter (30 days versus 12 months). What is important to remember is that such experiences might prevent people from using other services, thus perpetuating the vicious circle.

As a contributory factor to racial inequalities in oral health, discrimination based on race should be viewed as a social determinant of oral health [11]. However, what remains unclear from this line of research is why, how, and to what degree experiences with discrimination in everyday social settings, including a dental clinic or a hospital, influence the lives of racial minorities [2]. One popular explanation that the literature offers is based on the conceptual framework laid by the Major and O’Brien’s model of stigma-induced identity threat, which explains how subjective perceptions of discrimination within the healthcare system influence receipt of health services [21]. People may disengage from health services because of discrimination and insensitivity from healthcare staff (on the grounds of gender, race, culture, social class, sexuality or even symptom-related factors such as drug use or homelessness). They may feel alienated by negative discriminatory experiences; being subject to more paternalistic and coercive treatments and cultural or language barriers in assessments [33,34]. Collectively, this would lead to mistrust [19], non-compliance with treatment, and partial or complete disengagement from a dominant cultural institution such as healthcare, further attenuating the unmet need of care.

The current findings begin to fill an important gap in evidence regarding racial discrimination and the consequential emotional impact of discrimination on dental visits using quantitative data. In general, the uptake of dental services is conceptualized as issues of acceptability, affordability or availability, and contributory roles of the perceived systemic level and interpersonal discrimination are often overlooked. Dental visits could be a wider experience of racial discrimination prevailing in the society in general and in the healthcare system in particular. It is imperative to account for the accurate impact of discrimination and assess the contemporary policies that pave the way to racial discrimination. Our findings underscore the need to understand the patterns of discrimination and ways in which it influences engagement or disengagement from health services in a longitudinal framework. If understood, this could move policies forward and potentially facilitate developing effective strategies to deliver non-discriminatory and equitable healthcare services.

Few caveats should be acknowledged in interpreting the findings. First, the cross-sectional nature of the data limits the extent to which causal inferences can be drawn from our findings. Second, the single-item measure of perceived discrimination in healthcare pertained to experiences during the past 12 months, which intrinsically excluded people who did not seek care in the past year because of access-related issues. Third, the moderate response rate of the BRFSS might raise concerns about selection bias, although the probability weights used to correct for the differential probability of selection and non-response might have corrected it to an extent. Finally, the inconsistent adoption of the optional reactions to race module across the states limits the generalizability of our findings. Characteristics of the states in which the module was used may differ in significant ways from those of states that elected not to use it. Therefore, the present findings represent valid relationships between the variables of interest but cannot be viewed as representing the entire adult nation in the United States.

## 5. Conclusions

In a large population survey of adults in the United States, this study shows that the emotional impact of racial discrimination was associated with lower uptake of dental services. This association persisted even after accounting for various predisposing, enabling, and need factors. The preliminary findings from this study underscore the need to understand the patterns of discrimination and ways in which it influences engagement or disengagement from health services.

## Figures and Tables

**Table 1:** Characteristics of the sample and proportion of participants who visited the dentist in the past year by covariates (*n* = 11,950).

Explanatory Variables	All Sample		% with Dental Visit		*p* Value ^a^
	%	[95% CI]	%	[95% CI]	
*Gender*					<0.001
Male	52.8	[51.5–54.0]	64.3	[62.5–66.1]	
Female	47.2	[45.9–48.4]	72.8	[71.2–74.4]	
*Age group*					<0.001
18–24 years	10.7	[9.7–11.7]	62.1	[57.3–66.7]	
25–34 years	22.6	[21.4–23.8]	62.3	[59.3–65.2]	
35–44 years	22.3	[21.3–23.4]	69.4	[66.7–71.9]	
45–54 years	22.9	[21.9–23.9]	71.5	[69.2–73.7]	
55–64 years	17.2	[16.4–17.9]	73.6	[71.3–75.8]	
65+ years	4.3	[3.9–4.6]	71.6	[67.6–75.2]	<0.001
*Education*					
Less than high school	8.9	[8.0–9.9]	44.6	[38.8–50.4]	
High school	24.8	[23.7–25.9]	62.9	[60.4–65.4]	
Attended college	35.1	[33.8–36.3]	68.1	[66.0–70.2]	
College graduate	31.1	[30.1–32.1]	79.6	[78.0–81.1]	
*Annual income*					<0.001
Lowest	5.9	[5.2–6.6]	45.5	[39.5–51.6]	
2nd Lowest	15.1	[14.1–16.2]	49.1	[45.4–52.8]	
Middle	10.9	[10.1–11.8]	58.3	[54.2–62.4]	
2nd Highest	14.7	[13.8–15.6]	63.0	[59.6–66.3]	
Highest	53.2	[52.0–54.5]	79.8	[78.5–81.1]	
*Race/ethnicity*					<0.001
Non-Hispanic White	71.4	[70.2–72.6]	72.5	[71.1–73.8]	
Non-Hispanic Black	11.5	[10.7–12.5]	56.7	[51.8–61.4]	
Hispanic	11.0	[10.3–11.7]	57.0	[53.3–60.7]	
Asian/Others	5.1	[4.5–5.7]	62.3	[56.2–67.9]	
Multiracial	0.8	[0.6–1.1]	58.9	[43.7–72.6]	
*Missing teeth*					<0.001
No missing teeth	64.0	[62.7–65.1]	70.9	[69.4–72.4]	
One tooth or more	3.0	[34.8–37.2]	63.6	[61.4–65.6]	
*Smoking status*					<0.001
Current smoker	19.1	[18.0–20.2]	52.8	[49.6–56.1]	
Former smoker	22.8	[21.8–23.8]	70.8	[68.4–73.1]	
Never smoked	58.0	[56.8–59.3]	72.4	[70.8–73.9]	
*Health insurance*					<0.001
No insurance	12.2	[11.2–13.2]	36.3	[32.2–40.7]	
Insured	87.8	[86.7–88.7]	72.7	[71.5–73.9]	

^a^ Chi squared test was used for comparison.

**Table 2:** Crude associations between each indicator of racial discrimination and use of dental services in the past year among American adults (*n* = 11,950).

	% with Dental Visit		OR ^a^	[95% CI]	*p* Value
	%	[95% CI]			
*Healthcare discrimination*					<0.001
No	68.6	[67.4–69.8]	1.00	[Reference]	
Yes	54.2	[45.6–62.4]	0.57	[0.41–0.79]	
*Emotional impact of discrimination*					<0.001
No	68.9	[67.7–70.2]	1.00	[Reference]	
Yes	55.8	[49.7–61.7]	0.57	[0.44–0.73]	

^a^ Logistic regression was fitted and odds ratios (OR) reported.

**Table 3:** Regression models for the association between indicators of racial discrimination and use of dental services in the past year among American adults (*n* = 11,950).

**Healthcare Discrimination**	**Model 1A ^b^**		**Model 1B ^b^**		**Model 1C ^b^**	
	**OR ^a^**	**[95% CI]**	**OR ^a^**	**[95% CI]**	**OR ^a^**	**[95% CI]**
No	1.00	[Reference]	1.00	[Reference]	1.00	[Reference]
Yes	0.83	[0.58–1.17]	0.88	[0.62–1.25]	0.88	[0.62–1.25]
**Emotional Impact of Discrimination**	**Model 2A ^b^**		**Model 2B ^b^**		**Model 2C ^b^**	
	**OR ^a^**	**[95% CI]**	**OR ^a^**	**[95% CI]**	**OR ^a^**	**[95% CI]**
No	1.00	[Reference]	1.00	[Reference]	1.00	[Reference]
Yes	0.79	[0.60–1.03]	0.76	[0.58–0.99] *	0.76	[0.58–0.99] *

^a^ Logistic regression was fitted and odds ratios (OR) reported; ^b^ Model A was adjusted for predisposing factors (age, gender, race/ethnicity, education, income, and smoking status); Model B was additionally adjusted for enabling factors (health insurance); Model C was additionally adjusted for need factors (missing teeth); * *p* < 0.05.
